# Functional Magnetic Resonance Imaging Biomarkers Predicting Cognitive Progression in Parkinson Disease: Protocol for a Prospective Longitudinal Cohort Study

**DOI:** 10.2196/12870

**Published:** 2019-04-29

**Authors:** Brenda Hanna-Pladdy, Rao Gullapalli, Hegang Chen

**Affiliations:** 1 Diagnostic Radiology & Nuclear Medicine University of Maryland School of Medicine Baltimore, MD United States; 2 Epidemiology & Public Health University of Maryland School of Medicine Baltimore, MD United States

**Keywords:** Parkinson disease, cognition, disease progression, dementia, mild cognitive impairment, biomarkers, functional neuroimaging

## Abstract

**Background:**

Cardinal features of Parkinson disease (PD) are motor symptoms, but nonmotor features such as mild cognitive impairment (MCI) are common early in the disease process. MCI can progress and convert to dementia in advanced stages, creating significant disability and reduced quality of life. The primary pathological substrate for cognitive decline in PD is unclear, and there are no reliable biomarkers predicting the risk of conversion to dementia. A subgroup of PD patients with visual hallucinations may display more rapid conversion to dementia, suggesting that regional markers of visuoperceptual dysfunction may be sensitive to pathologic density in posterior cortical regions.

**Objective:**

The purpose of this project is to characterize PD-MCI and evaluate the utility of genetic and neuroimaging biomarkers in predicting cognitive outcomes with a prospective longitudinal study. We will evaluate whether accelerated cognitive progression may be reflected in biomarkers of early posterior cortical changes reflective of α-synuclein deposition.

**Methods:**

We will evaluate a cohort of early-stage PD patients with the following methods to predict cognitive progression: (1) serial neuropsychological evaluations including detailed visuoperceptual functioning across 4 years; (2) genetic analysis of *SNCA* (α-synuclein), *MAPT* (microtubule-associated tau), and *APOE* (apolipoprotein E); (3) an event-related functional magnetic resonance imaging paradigm of object recognition memory; and (4) anatomical and regional brain activation changes (resting-state functional magnetic resonance imaging) across 4 years.

**Results:**

The project received funding from the National Institutes of Health in August 2017, and data collection began in February 2018. Enrollment is ongoing, and subjects will be evaluated annually for 4 years extended across a 5-year project including data analysis and image processing.

**Conclusions:**

Cognitive, genetic, and structural and functional magnetic resonance imaging will characterize neural network changes predictive of cognitive progression in PD across 4 years. Identification of biomarkers with sensitivity for early prediction and estimation of risk for conversion to dementia in PD will pave the way for effective intervention with neuroprotective therapies during the critical stage when treatment can have the greatest impact.

**International Registered Report Identifier (IRRID):**

DERR1-10.2196/12870

## Introduction

### Background

Parkinson disease (PD) is the second most common neurodegenerative disorder characterized by progressive movement deficits and is caused by degeneration of nigrostriatal dopamine neurons and deposition of α-synuclein in intraneuronal Lewy body (LB) inclusions [1]. Nonmotor features can characterize the earliest phase of the disease even before clinical motor impairment. Mild cognitive impairment (MCI) is a prominent nonmotor feature that is present early in the disease process and increases over time, eventually converting to dementia (Parkinson disease dementia [PDD]). Follow-up studies reveal that MCI is present in one-third of patients and progresses to PDD, with conversion greater than 80% after 15 to 20 years [2,3]. Prospective longitudinal studies demonstrate that PDD is common, with a 4-year prevalence 3 times higher than the general population [4,5]. However, dementia onset is variable, with a subset of patients demonstrating rapid conversion, resulting in significant disability and poor quality of life [4,6,7]. The neural and pathophysiologic mechanisms predicting rates of PD progression remain poorly understood and are key research priorities. Biomarkers signaling risk for PDD will facilitate intervention during early therapeutic windows, thus optimizing the chances of slowing progression.

Early cognitive deficits in PD have been characterized as executive and considered to be related to reductions in frontostriatal activity, suggesting a dopaminergic substrate [8]. However, characterization of MCI in early PD has revealed extensive deficits outside the executive domain and is not fully explained by dopamine depletion alone [9,10]. Therefore, identification of early reliable biomarkers that are predictive of risk for dementia conversion will require models of PD-MCI that focus on markers outside of the dopaminergic network. Widespread distribution of dopamine receptors in the neocortex is well recognized, with a rostrocaudal dopamine receptor gradient with the highest receptor densities in the prefrontal cortex and lowest densities in the occipital regions [11]. Therefore, biomarkers targeting posterior cortical changes may be more reliable for earlier prediction.

There is growing evidence of the presence of extranigral pathology that occurs before dopamine depletion. Multiple pathologies are linked to cognitive deficits in PD, including cholinergic, dopaminergic, and diffuse cortical LBs associated with α-synuclein, tau, and Alzheimer disease (AD)–like lesions of amyloid-β plaques [12,13]. PD shares a common neuropathological pattern to LBD and overlaps partially with AD, complicating early diagnosis based on cognitive profiles [14]. Although the spread of α-synuclein is the strongest correlate of PDD, up to 50% of patients also develop sufficient amyloid-β plaques and tau-containing neurofibrillary tangles for a secondary diagnosis of AD [15]. Thus, these pathologies may act synergistically to confer a worse prognosis.

Patients with parkinsonism with cognitive deficits in the first year are diagnosed with Lewy body disease (LBD), whereas those with cognitive deficits after motor deficits are diagnosed with PDD. Although the main features of PDD and LBD are similar (visual hallucinations, visuospatial deficits, attention and executive deficits, and variable memory deficits with parkinsonism), controversy still exists as to whether PDD and LBD are distinct entities. It is conceivable that they represent 2 points on a continuum, with the type and degree of concurrent pathology influencing the timing and rate of cognitive decline [16]. LB inclusions in neocortical and paralimbic regions in PD are predictive of the rate of cognitive decline and memory and visuoconstructional deficits [14]. This implies that the density of α-synuclein and concurrent amyloid-β could act as modulators in the relative timing of cognitive decline and may predict rates of cognitive progression [1]. This is supported by parietal and superior temporal cortical atrophy in early PD-MCI relative to late-onset cognitive decline in PD, even in the absence of cognitive differences [17]. This suggests that pathological burden in parietotemporal regions is greater in PD with early-onset cognitive decline [18]. To advance therapeutics targeting nonmotor features and determine whether PDD and LBD are distinct disorders, it is essential to determine whether the pathology responsible for early deficits also has a synergistic role in dementia conversion.

Current studies looking at different types of biomarkers suggest different possible contributors to PDD, but findings have been inconsistent across studies [19]. Understanding the relationships between genetic risk factors for distinct pathologies and their resultant clinical presentation in PD is crucial for the development of disease-modifying interventions. Evaluating early cognitive deficits in PD and markers of future cognitive progression will shed light on whether PD pathology progresses in a consistent, expected pattern. Prospective longitudinal studies are essential to identify the initial cognitive symptoms and temporal pattern of atrophy and neural progression predictive of outcomes [7,20] *.* Thus, based on the models of pathological staging and anticipated sequence resulting in cognitive decline, accelerated cognitive progression may be reflected in biomarkers of early posterior cortical changes reflective of α-synuclein [12].

Cognitive profiles provide an index of staging of neuroanatomic regions, reflecting density and distribution of pathologic burden. Symptoms of visual hallucinations are associated with posterior cortical involvement and predict 75% conversion to PDD in 2.5 years, with widespread atrophy providing critical clues for biomarker identification [21]. Although visual hallucinations are specific to cognitive decline in PDD, they are not sensitive, given the low incidence of reporting by patients. Thus, the detection of associated visuoperceptual deficits may improve prediction of posterior cortical pathology and future cognitive decline.

### Objectives

The long-term objectives of this study are the investigation of the predictive validity of early cognitive profiles in PD and identification of genetic and magnetic resonance imaging (MRI) markers signaling more rapid conversion to dementia. In this study, we will target the posterior cortex as a reliable marker of pathologic burden based on the proposed caudal-rostral progression of α-synuclein and the rostrocaudal dopamine gradient, with lower occipital receptor density eliminating the effects of dopamine replacement therapy (DRT). Our hypothesis, based on the models of pathologic staging, is that earlier involvement of posterior cortical regions and the dorsal and ventral visual pathways (with or without visual hallucinations) are reliable markers for cognitive progression. To date, there have been few longitudinal and MRI studies focused on biomarker identification signaling cognitive progression in PD. Neuroimaging techniques offer promise in detecting neuronal changes before performance decrements and identifying biomarkers of accelerated cognitive progression.

## Methods

### Overview

The primary objective is to investigate the prognostic value of PD-MCI subtypes and genetic variation in predicting the relative risk for progression to dementia utilizing a prospective longitudinal cohort followed for over 4 years. Tables 1 and 2 provide a detailed screening assessment and dependent study measures and timeline. The objectives will be addressed through the following methods: (1) neuropsychological evaluations and novel visuoperceptual tasks will be given annually for 4 years to nondemented PD patients (at baseline), and the predictive validity of visuoperceptual cognitive deficits relative to memory and executive deficits and ideal cutoffs (−1.5 vs −2 SD) for reliable prediction will be determined; (2) an exploratory genetic analysis of how variations in the *SNCA* (α-synuclein), *MAPT* (microtubule-associated tau), and *APOE* (apolipoprotein E) genes influence cognitive progression will be conducted; (3) the utility of task-activated functional magnetic resonance imaging (fMRI) as a probe for the risk for cognitive progression will examine altered posterior cortical networks before clinical manifestation; and (4) the anatomical and regional brain activation patterns predictive of cognitive progression will be determined.

Screening and recruitment will be conducted as outlined with year of testing denoted by 1-4. If we have attrition related to completing the MRI protocol or patients do not return for years 2-4, we will recruit additional patients in year 3 to ensure adequate numbers of patients and converters. The fifth year will be utilized for completion of data collection to account for attrition, image processing, and data analysis.

### Patients

Nondemented idiopathic PD patients will be recruited and evaluated in the *on* medication state. The severity of PD will be graded by the Unified Parkinson Disease Rating Scale (UPDRS-III) motor section [22]. Subjects will be asked to provide consent in accordance with Institutional Review Board (IRB) policies and protected by Health Insurance Portability and Accountability Act regulations, and IRB approval has been obtained from the University of Maryland Baltimore (UMB), School of Medicine. We will recruit 120 PD patients who will be evaluated annually for 4 years on neuropsychological and MRI measures in a 5-year study. Patients will be recruited from the University of Maryland School of Medicine as well as through other advertisements to recruit patients from surrounding areas. Our goal is to complete 100 patients, so we will overrecruit because of anticipated attrition. Potential subjects will be screened, and information regarding the Unified Parkinson’s Disease Rating Scale (UPDRS), mood, Activities of Daily Living (ADLs), and Montreal Cognitive Assessment (MoCA) will be acquired to determine the study eligibility [22,23]. The principal investigator is a clinical neuropsychologist who will review the study and the inclusion criteria and will confirm the diagnosis with the study neurologist who will examine all the patients.

### Inclusion and Exclusion Criteria

General and specific inclusion and exclusion criteria for both PD patients and controls are reviewed below.

General criteria: (1) age 50 to 77 years to avoid early-onset PD and (2) no evidence of depression on the Beck Depression Inventory (BDI-II >18 is predictive in mild PD) [24].

PD criteria: (1) Diagnosis of idiopathic PD based on the presence of 2 cardinal features [25], (2) improvement with DRT, (3) minimal or mild disease severity based on the Hoehn and Yahr rating scale of <2.5 and motor UPDRS <30 *on* DRT medications [22], and (4) normal or MCI cognition based on MoCA screening (>21) at baseline [26].

### Exclusion Criteria

(1) Other neurologic disorder or stroke; (2) dementia (MoCA <21 at baseline) or functional decline on instrumental ADLs related to cognitive deficits [27]; (3) major psychiatric disorder, including alcohol or substance abuse; (4) concurrent, unstable or serious medical condition; (5) major head trauma; (6) chronic use of psychoactive medications; (7) significant dyskinesia on neurological examination; (8) tremor greater than 2 for upper extremities and head tremor greater than 1 based on the MDS-UPDRS on neurological examination; and (9) claustrophobia, pacemaker, neurostimulator, or other implants or other factors that interfere with the ability to lay still in the MRI scanner for 1 hour.

### Timeline for Schedule of Assessments

Study subjects will be enrolled and evaluated annually across 4 years (see Table 2 for a detailed timeline of enrollment and schedule of assessments). Neuropsychological, behavioral, and neurological assessments, including the MD-UPDRS motor section III, will be conducted annually. Genetic samples and cognitive activation paradigm will only be conducted at year 1 and utilized as predictors of 4-year outcomes, whereas the remaining MRI measurements will be conducted annually (Table 2).

#### Assessments

##### Neuropsychological Assessment

The battery was selected based on the identified PD cognitive deficits and task force recommendations. The battery will include estimates of premorbid verbal intellectual functioning (American New Adult Reading Test) [28], measures of both verbal and nonverbal memory and attention, and several executive tasks. Memory tests will use equivalent alternative forms across years 1 to 4 to control for practice effects [29].

**Table 1:** Screening, assessment, and dependent study measures.

Measures	Individual tests
Genetic markers	*SNCA* (α-Synuclein), rs356219
	*MAPT* *H1/H2* haplotypes (microtubule-associated tau), rs1800547
	*APOE ɛ2/ɛ3/ɛ4* alleles (Apolipoprotein E), rs429358 and rs7412
Screening	Unified Parkinson Disease Rating Scale Motor Section III
Behavioral measures	Montreal Cognitive Assessment
	Beck Depression Inventory II
	Edinburgh Handedness Inventory
	Beck Anxiety Inventory
	The Parkinson Disease Sleep Scale
	The Parkinson Disease Questionnaire
	Neuropsychiatric Inventory of Psychopathology in Dementia
	Adelaide Activities Profile
Neuropsychological assessment	American New Adult Reading Test
	Digit Span and Letter Number Sequencing
	Phonemic and Semantic
	Hopkins Verbal Learning Test-Revised
	Benton Visuospatial Memory-Test Revised
	Boston Naming Test
	Judgment of Line Orientation
	Visual Object and Space Perception
	Visual Form Discrimination
	Stroop Color and Word Test
	Delis Kaplan Executive Function Scale Trails 1-5
	Delis Kaplan Executive Function Scale Tower Test
	Computerized Version of the Wisconsin Card Sorting Test
	Finger tapping
	Grooved pegboard
Neuroimaging	Cortical thickness; Voxel-based morphometry
	resting-state fMRI; Diffusion Tensor Imaging
	Task-activated fMRI imaging paradigm—object memory (encoding, immediate and delayed memory recall)

**Table 2:** Timeline of enrollment and schedule of assessments.

Data Collection Measures	Year 1	Year 2	Year 3	Year 4
Screening and recruitment	X^a^	X	—^b^	—
Unified Parkinson Disease Rating Scale -III (motor section)	X	X	X	X
Saliva samples for genetics	X	—	—	—
Neuropsychological and behavioral measures	X	X	X	X
fMRI Cognitive activation paradigm	X	—	—	—
Structural MR, rs-fMRI & DTI	X	X	X	X

^a^Denotes time points when data collection is active.

^b^Not applicable.

##### Attention

Visual attention will be evaluated with the Stroop Color-Word test [30], whereas auditory working memory will be evaluated by the Letter-Number Sequencing and auditory Digit Span backward and forward from the Wechsler Adult Intelligence Scale–Fourth Edition [31].

##### Executive Tasks

A computerized version of the Wisconsin Card Sorting Task [32] and the Delis Kaplan Executive Functions Scale (Trails and Tower of London) with demonstrated reliability and validity in measuring executive functions will be given [33].

##### Language

Language will be assessed by the Boston Naming Test [34] and phonemic and semantic fluency.

##### Memory Tasks

The Hopkins Verbal Learning Test [35,36] for verbal memory and the Brief Visuospatial Memory Test [37] will both be utilized in alternative forms across the years*.*

##### Sensorimotor Tasks

Motor tasks of speed, precision, and dexterity (finger tapping and grooved pegboard) will be evaluated bilaterally [38].

##### Other Measures

Other measures include evaluation of hallucinations (Neuropsychiatric Inventory [39] and thought disorder item UPDRS-TD [40]), mood and anxiety (BDI-II; Beck Anxiety Inventory) [41,42], quality of life (The Parkinson Disease Questionnaire-39]) [43], and ADLs with the Adelaide Activities Profile, which focuses on instrumental activities [27].

##### Visuoperceptual Tasks

The Judgment of Line Orientation (JLO) task and Benton Visual Form Discrimination tasks will measure visual orientation [44] and visuoperceptual discrimination [45]. An abbreviated version of the Visual Object and Space Perception battery will be given to evaluate silhouettes presented in unusual perspectives and progressive silhouettes as well as decision of objects presented in rotated manners [46]. On the basis of the tasks previously developed in our laboratory in early PD, subjects will be evaluated on *visuoperceptual judgments of equidistance* (judging line lengths and width intervals). Presentation software (Neurobehavioral Systems) [47] will present the perceptual stimuli (4-second presentation). The first task requires *length estimation* of 2 lines in either a vertical or a horizontal orientation and evaluation of whether they are the *same* or *different*. The second task, *width interval estimation*, compares 2 interval spaces between 3 horizontal lines to determine if they are the *same* or *different*. Subject responses will be entered as same, different, or a miss (failure to respond) by the experimenter. Each task contains 24 trials, randomized for each subject and varied by 3 line lengths (4, 8, and 12 cm) and 4 levels of difficulty (same, easy, difficult, and very difficult).

### Genotyping

Genetic variations will be explored relative to cognitive and neuroimaging progression and 4-year outcomes to determine the association with cognitive progression. Genotyping for *SNCA* rs356219, *MAPT* H1/H2 haplotypes (rs1800547) *,* and *APOE ɛ2/ɛ3/ɛ4* alleles (rs429358 and rs7412) will be conducted using DNA extracted from saliva and performed with TaqMan assays. TaqMan single nucleotide polymorphism assays will extract DNA from saliva samples, and they have been demonstrated to yield comparable results to blood samples but eliminate the need for an invasive procedure, reduce risk, and increase recruitment. To ensure optimal analysis and avoid false-positive amplifications, at least 2 no-template controls will be used per assay. Assays will be conducted by the UMB Center for Innovative Biomedical Resources genetics core facilities.

### Imaging Protocol

**Figure 1:**
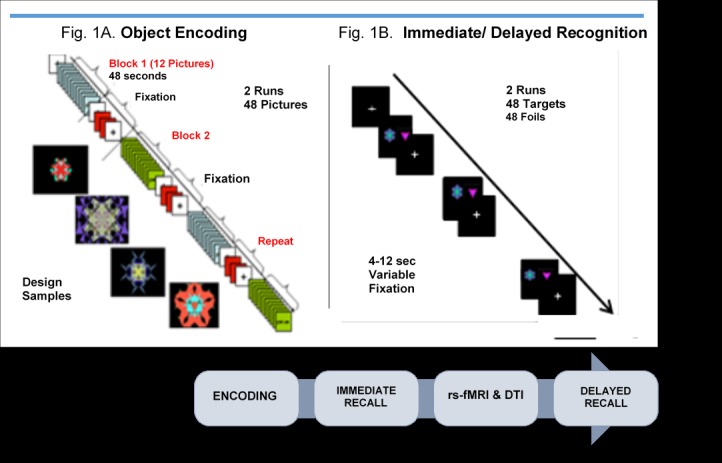
Cognitive activation memory paradigm. rs-fMRI: resting-state functional magnetic resonance imaging; DTI: Diffusion Tensor Imaging.

Imaging is performed on a Siemens PRISMA 3T MRI scanner using a 64-channel receive head coil. After obtaining scout images in all 3 planes, a high-resolution volumetric sagittal T1-weighted-MPRAGE (TE=2.94 ms, TR=2300 ms, TI=900 ms, flip angle=9^°^, 1-mm isotropic resolution) is obtained, which serves as the anatomical reference from which regional and whole-brain volumes and cortical thickness are extracted. The total acquisition time for MPRAGE is 5 min and 30 s. Furthermore, 6 sets of fMRI data are then obtained in 2 phases. During the first phase, 2 encoding runs are performed, followed by 2 immediate recall acquisitions. After a delay of 18 min, the second phase consisting of 2 delayed-recall fMRI acquisitions is obtained (see Cognitive Activation Paradigm, Figure 1). Data for fMRI are obtained using a 2-dimensional multiband single-shot T2*-weighted, echo-planar imaging sequence (TE=30 ms, TR=2000 ms, Flip 90^°^, 2-mm isotropic resolution) with a multiband acceleration factor of 4 covering the entire brain in the axial plane [48-50]. Between the immediate-recall and delayed-recall sessions, both diffusion tensor data and rs-fMRI data are acquired. The diffusion tensor images are obtained using a multiband double-refocused spin-echo echo-planar imaging sequence (TE=78 ms, TR=3000 ms) using b-values of 0 and 2500 s/mm^2^ and uniformly sampling the sphere in 136 directions with 5 b=0 acquisitions at an isotropic resolution of 2 mm for a total acquisition time of 7 min and 19 s. rs-fMRI data are obtained using a T2* gradient echo EPI sequence (TE=30 ms, TR=2000 ms; 300 volumes) also at an isotropic resolution of 2 mm for a total acquisition time of 10 min and 12 s. In addition, fast spin echo-based T2-weighted images (TE=100 ms, TR=5340 ms) and FLAIR images (TE=83 ms, TR=9000 ms; TI=2500 ms) are also obtained.

fMRI and rs-fMRI data will be preprocessed using both AFNI and SPM. Preprocessing includes slice timing correction, registration of all the volumes to the first volume of the time series, spatial blurring (6 mm FWHM), and band-pass filtering (0.01 Hz<f<0.1 Hz). Functional MRI data will be spatially normalized to a common Montreal Neurological Institute (MNI) template. The global signal from the mean BOLD time series from the whole brain mask and the 6 motion-correction parameters will be used as regressors in the model to remove the variance related to nonneuronal contributions and motion.

### Cognitive Activation Paradigm

An fMRI event-related object memory task using fractal images will measure BOLD response in PD patients at baseline. Stimuli are based on the object and spatial memory study by Floel et al [51]. Stimuli will be presented on a screen and synchronized with the scanner via an optic relay triggered by the radiofrequency pulse, and responses will be acquired through Presentation software. The paradigm will consist of 6 BOLD sequences (2 for encoding, 2 for immediate recall, and 2 for delayed recall), but with an 18-min delay when structural and rs-fMRI data are acquired (Figure 1). During object encoding, subjects will view 48 images presented in a fixed block across 2 runs. Each run will include 24 designs presented across 2 trials in 4 blocks of 12 images. Stimulus presentation is 4 seconds for each image (48 secs for each block), followed by 2 baseline fixations separated by repeating 2 figures for 6 times (96 secs) between each of the blocks. Each run will last 8 min and 30 seconds for the encoding condition.

An event-related fMRI paradigm will evaluate immediate and delayed recognition of the 48 designs based on discrimination from 48 foils. Each of the recognition trials will consist of 2 runs with variable 4- to 12-second fixations between each stimulus presented for 4 seconds (runs last 7-8 min and 32 secs). Stimulus presentation order and interstimulus intervals will be randomized across 14 different experiments. Subjects will be instructed to look at the designs presented on the screen and recall whether the designs are old designs (targets) or new designs (foils). Responses and reaction times will be recorded by pressing the key press with their index finger for *yes* or pressing their middle finger for *no*. Data will be analyzed separately for *accurate*, *inaccurate*, and *missed responses*. Following encoding, the immediate recognition runs will be collected. An 18-min delay when structural and rs-fMRI are conducted will precede the delayed recognition recall condition.

### Resting-State and Voxel-Based Morphometry

#### Cortical Thickness

MPRAGE images will be processed using the pipeline established in Free Surfer [52]. Cortical thickness from various regions of interest (ROIs) is then determined as the mean of difference between WM and pial surfaces. We will generate an average surface map to which each surface map will be aligned followed by generation of a group difference map.

#### Connectivity Analysis

Following the preprocessing steps as described earlier, we will register all the 300 volumes collected for rs-fMRI to the first volume of the time series. As differential head motion between groups can introduce artifacts, we will equate the PD and control groups with a *scrubbing* procedure to remove volumes compromised by motion [53]. The resting-state series will then be registered to the high-resolution structural images, spatially normalized to standard space using the MNI atlas, and resampled to a spatial resolution of 2.0 mm isotropic and then spatially blurred using a 5-mm Gaussian kernel. The segmented masks (GM, WM, and CSF) created as part of the measurement for cortical atrophy will account for time series variance from the nonneuronal contributions of CSF and WM. In addition, the 6 standard movement parameters from registration will be regressed out of the 4-dimensional data. Graph-theoretic analysis on selected ROIs that are relevant to PD cognition based on our hypotheses will be performed and expanded to a more global approach using Graph Analysis Toolbox (GAT) [54]. The ROI method will consider regions such as *caudate, putamen, cingulate, orbitofrontal, dorsolateral prefrontal cortex (DLPFC), superior and inferior parietal, posterior and anterior visual cortex, superior and medial temporal.* Pairwise correlations between mean BOLD time series using ROIs will be utilized to create functional connectivity matrix followed by the Fisher transformation to z score association matrix. Various topological features of the networks will be captured at the global and regional level such as total connectivity, normalized clustering coefficient, normalized path length, density, global efficiency, and betweenness centrality [55]. Between-group comparisons will be made using a 2-sample *t* test after correcting for multiple comparisons with significance set at *P*<.05. To determine the relationship of the network topology with cognition, we will correlate all subtests of the neuropsychological and visuoperceptual assessments using the Pearson correlation coefficient comparing PD with normal cognition across 4 years and PD with cognitive progression.

Using a more global approach, we will perform cortical/subcortical parcellation to define 200 brain regions to identify and understand the influence of other networks on PD network topology and to assess whether taking such interactions into account influences predictions in PD outcomes. The extracted time courses from each of these regions will be correlated against one another to produce a 200×200 adjacency (correlation) matrix, followed by the Fischer transformation to z score association matrix. Similar metrics will be extracted as in the case of the limited ROIs described above.

Activation maps from fMRI will be analyzed using statistical parametric methods, and contrasts will be conducted using multiple regression with the General Linear Model, allowing for multiple predictors to be built into the model [56]. Regressors representing the experimental conditions of interest will be modeled with a hemodynamic response function and entered into the multiple regression analysis using both fixed- and random-effects models. Whole-brain statistical analyses will be conducted, and statistical parametric maps will be generated to examine brain regions that differ in activation between the groups and conditions (encoding and immediate and delayed recall). These analyses will be performed in each group separately using a repeated-measures analysis of variance model and then between groups (PD and PD-MCI). We will inspect the entire brain for areas of activation, and areas will be considered significant if they exceed a threshold of q<.001 (False Discovery Rate). We will evaluate the relationship between brain activation and cognition inside and outside the scanner.

### Statistical Analysis and Power

A sample of 120 PD patients is planned for this study. On the basis of the experience of longitudinal studies in PD, the attrition (mortality and loss to follow-up) is anticipated at 16% for 4 years. Thus, initial recruitment of 120 patients will yield about 100 subjects after 4 years. On the basis of published data, we estimate 70% PD-NC and 30% PD-MCI patients in our sample, and conversion rates of 60% from NC to MCI and 80% from MCI to PDD after 4 years. One of our primary goals is to evaluate progression patterns to determine time-specific differences between groups (NC vs MCI at baseline and PDD vs MCI, MCI vs NC, and PDD vs NC at year 4). Our study will have 80% power to detect medium-to-large average differences across time between the different groups under various within-subject correlations. In terms of predictive models for 4-year outcomes, our study will have 90% power to detect at least 0.86 area under the receiver operating characteristics curve (AUROC) for PDD conversion and 95% power to detect at least 0.74 AUROC for MCI at year 4.

To study differential cognitive progression patterns, generalized estimating equations will be used for describing the longitudinal trajectories of neuropsychological and visuoperceptual performances between PD-NC and MCI groups comparing baseline performance with different time points. The model will provide estimates of PD-MCI versus PD-NC differences at specific time points and longitudinally. Covariates such as age, UPDRS, and visual illusions or hallucinations will be added to the model to enable determination of adjusted differences.

We will investigate the prognostic value of baseline cognition in predicting relative risk for progression to PDD through a multiple logistic regression model. The best combination of baseline measures and covariates included in the prediction model will be selected by the elastic net procedure. Summary statistics of the AUROC will be used to evaluate the predictive power. Sensitivity, specificity, positive predictive value, and negative predictive value will be calculated from the optimal threshold determined by the Youden index. The prediction models will be cross-validated by training and testing methodology.

## Results

The project received funding from the National Institutes of Health in August 2017, and data collection began in February 2018. Enrollment is ongoing, and subjects will be evaluated annually for 4 years extended across a 5-year project including data analysis and image processing.

## Discussion

### Overview

Cardinal features of idiopathic PD include motor symptoms attributed to dopaminergic depletion of the dorsal striatum. Nonmotor features of the disease such as cognitive impairment are common early in the disease process and can dominate in advanced stages, creating significant disability and poor quality of life. Follow-up studies reveal that MCI is present in one-third of the patients and can progress at variable rates to PDD. After 15 to 20 years, more than 80% of patients with MCI are diagnosed with PDD [2]. However, the neural and pathophysiologic mechanisms underlying variable cognitive deficits and their progression in PD remain poorly understood. Cognitive profiles in nondemented early-stage PD can be widespread, extending beyond expected frontostriatal circuitry. Furthermore, DRT can have a paradoxical effect on attention and memory, with evidence for overdosing of nondepleted ventral striatal circuits early in the disease process, suggesting that these deficits may be unreliable predictors of cognitive progression [9,10]. PD-MCI is heterogeneous in clinical presentation (executive, memory, and visuospatial), reflecting the presence of extranigral pathology in addition to dopamine. The co-occurrence of LB inclusions (α-synuclein) and Alzheimer pathology (amyloid-β and tau) is essential for the development of PDD, but the neurobiological basis of PD-MCI is unknown. Density of amyloid-β is predictive of neocortical atrophy, but how the predominate pathology in PDD (α-synuclein) influences clinical features or signals conversion to dementia is unclear.

Our central hypothesis, based on models of pathologic staging, is that earlier involvement of posterior cortical regions and the dorsal and ventral visual pathways (with or without the presence of visual hallucinations) are reliable markers for future cognitive progression. Our strategy toward this overarching goal is to characterize cognitive, genetic, and neural network progression in PD over a 4-year period. This allows evaluation of variable timing of onset and accelerated rates of cognitive progression, reflecting a deviation from expected stages of progression. Prospective longitudinal studies are essential to identify the initial cognitive symptoms and temporal pattern of atrophy and neural progression predictive of outcomes. These studies have been lacking, with overreliance on cross-sectional motor and pathological dementia studies in PD [7,20].

Our project is built on the most recent advances in scientific understanding of cognitive, genetic, and neural markers of PD progression. We will translate this information to clinical applications through the development and validation of novel cognitive and neuroimaging markers that signal onset of PDD. Although the visuoperceptual and posterior cortical changes may not predict conversion in all patients, our model allows simultaneous measurement of other cognitive domains and associated brain regions, thus allowing verification of alternative hypotheses and combinations of cognitive domains.

### Cognitive Progression in Parkinson Disease

The Movement Disorder Task Force guidelines for the diagnosis of PD-MCI are focused on identifying the earliest stage of cognitive impairment by providing PD-specific criteria [7,20]. The heterogeneity in PD-MCI is recognized, with nonamnestic subtype as the most common, but few studies have evaluated PD-MCI subtype and conversion to PDD. In the few studies conducted, many different subtypes have been implicated [20]. In a 5-year longitudinal study, posterior cortical cognitive deficits were associated with PDD [57]. We aim to establish whether early cognitive and genetic profiles and neural network changes before clinical manifestation can be used for the estimation of pathological burden to assess risk for rapid cognitive progression.

### Genotyping Cognitive Risk in Parkinson Disease

There is contradictory evidence regarding α-synuclein levels in PD and how expression levels might be modified by genetic variability [58]. As blood α-synuclein levels may not be sensitive early in PD, measures of genetic variation serving a regulatory role might be useful for early prediction [59]. Variation in the SNCA gene has demonstrated influences on blood and brain and likely influences the expression of α-synuclein in PD expressed by genotype rs356219 [58,59]. Although APOE status does not appear to be associated with PD [60] and findings have been inconsistent regarding PDD, 2 recent studies reported that the APOE *ɛ4* allele predicted cognitive deficits in PD [61]. The MAPT H1 haplotype is a well-known risk factor for PD, but there have been contradictory findings as to whether it is a risk for rapid cognitive progression in PD [61]. Genetic variation impacts the age of onset and disease risk, with *SNCA* and *MAPT* genes contributing to progression and cognitive impairment [62]. There is also racial genetic variation influencing the risk of PD and presentation, although genetic variation impacting cognition has not been well investigated.

### Task-Activated Functional Magnetic Resonance Imaging

This study will evaluate the utility of task-activated fMRI as a probe for the risk of cognitive progression by examining altered posterior cortical networks before clinical manifestation. It remains unclear whether PD-MCI profiles confer a higher risk of conversion to PDD. Frontal executive and posterior cortical cognitive functions have been considered as risks for conversion, whereas the amnestic memory has been a focus in AD prediction. Combining cognitive and neural biomarkers may improve the prediction of conversion to PDD [63]. Task-activated fMRI offers promise for detecting activations during cognitive tasks to identify the salient aspects of subclinical impairment that are predictive of cognitive outcomes. There have been few fMRI biomarker investigations in PD, although 1 study identified overactivation in DLPFC and posterior parietal regions on a subclinical visuospatial task [64]. However, the predictive nature of these findings is unclear. We have developed a novel fMRI paradigm that targets posterior cortical regions through visuospatial processing and also activates memory regions, allowing for the possibility of more than 1 cognitive profile predicting conversion. BOLD patterns during encoding and immediate and delayed recall of task-activated nonverbal memory will evaluate dorsolateral prefrontal cortex (executive), medial temporal (memory), and occipito-parieto-temporal (visuospatial) activations as predictors for 4-year outcomes. We predict that greater percentage signal change in occipito-parieto-temporal regions during a visuospatial performance at baseline will predict cognitive progression in multiple cognitive domains.

### Anatomical and Regional Brain Activation Patterns Predictive of Cognitive Progression

Significant atrophy has been associated with transition from MCI to AD [65,66]. However, there is limited work on cortical thinning in early PD, and no longitudinal studies have evaluated cortical thickness measures as biomarkers signaling rapid cognitive progression in PD [17,67]. Critically important to prediction is evaluating differences in patterns of cortical atrophy based on the timing of onset of cognitive progression. This could reveal an anatomical basis for the timing of cognitive decline, thus elucidating the pathological burden in PD [17].

There is an increased interest in studying resting-state brain networks to try and understand interactions between disparate brain regions. Rs-fMRI measures the strength of functional interactions between brain regions based on temporal correlations of the spontaneous fluctuations in the resting-state BOLD signal, such that synchronous temporal changes in signal are functionally connected [68]. Rs-fMRI has been used to test the efficiency and strength of large-scale neuronal networks in the absence of a task. Although rs-fMRI has revealed the natural history of amnestic MCI to AD, very little is known about its efficacy as a biomarker in PD [69,70]. Characterizing differences in PD will elucidate temporal changes in functional networks related to relative changes in frontal, medial-temporal, and posterior cortical regions with cognitive progression and provide neural insights into the timing of pathophysiological changes in PD.

Cortical thickness, voxel-based morphometry, and rs-fMRI will characterize the evolution of regional brain degeneration annually for 4 years in PD patients. We predict a higher rate of cortical thinning and decreased functional connectivity in occipito-parieto-temporal regions in PD patients with cognitive progression.

### Conclusions

This project will incorporate specific areas of innovation, including the use of predictive models; inclusion of novel fMRI biomarkers; segregation of motor, executive, and visuoperceptual task demands; and conceptualization of PD progression based on nonmotor features. Making predictions is a critical task in translational medicine and is of great importance for diagnosis, prognosis, and prediction of treatment response [71,72]. Our predictive model will provide a unique contribution by using cognitive, genetic, and neuroimaging markers to characterize cognition in PD. Previous neuroimaging investigations have focused on PET and SPECT, and there have been few task-activation paradigms developed for PD. The proposed project is innovative in establishing a combined cognitive and fMRI approach for early identification of risk for cognitive progression and to establish the phenotypes of PD connectome using graph theoretic analysis. The proposed project will contribute to a new conceptualization of neurodegeneration in PD. This new framework will include variable rates of onset and progression of both motor and nonmotor features, with recognition that pathology can deviate from expected stages and have accelerated progression.

### Summary

There are significant challenges in PD biomarker development with potential to advance therapeutics, in particular, related to nondopaminergic responsive features of PD. Study findings will address these challenges and provide insight into pathophysiological mechanisms by characterizing heterogeneity in cognitive, genetic, and neural temporal progression. This will contribute to an altered conceptualization of PD and LBD neurodegeneration based on the timing of onset and progression of cognitive deficits relative to structural and functional changes. Currently, there is no reliable biomarker predictive of risk for rapid cognitive decline, and there have been few longitudinal studies of temporal progression. Identifying reliable biomarkers will aid in the early and accurate prediction of risk for PDD and provide objective monitoring and response to treatments in the earliest stage when therapies have their greatest impact. Identification of patients at risk for early cognitive progression will have a high impact on patient management strategies and will facilitate intervention at the earliest stage when treatments can have their greatest impact on progression. Study results will provide a foundation for the development of disease-modifying agents and preventative clinical trials.

## Abbreviations

AD: Alzheimer disease
ADLs: Activities of Daily Living
AUROC: area under the receiver operating characteristics curve
BDI: Beck Depression Inventory
DLPFC: dorsolateral prefrontal cortex
DRT: dopamine replacement therapy
fMRI: functional magnetic resonance imaging
IRB: Institutional Review Board
LB: Lewy body
LBD: Lewy body disease
MCI: mild cognitive impairment
MNI: Montreal Neurological Institute
MoCA: Montreal Cognitive Assessment
MRI: magnetic resonance imaging
PD: Parkinson disease
PDD: Parkinson disease dementia
ROIs: regions of interest
rs-fMRI: resting-state functional magnetic resonance imaging
DTI: Diffusion tensor imaging
UPDRS: Unified Parkinson Disease Rating Scale
